# Structural and Kinetic Characterization of Hyperthermophilic NADH-Dependent Persulfide Reductase from *Archaeoglobus fulgidus*

**DOI:** 10.1155/2021/8817136

**Published:** 2021-03-09

**Authors:** Sherwin Shabdar, Bukuru Anaclet, Ana Garcia Castineiras, Neyissa Desir, Nicholas Choe, Edward J. Crane, Matthew H. Sazinsky

**Affiliations:** ^1^Department of Biology, Pomona College, 175 West 6th Street, Claremont, CA 91711, USA; ^2^Department of Chemistry, Pomona College, 645 N. College Ave., Claremont, CA, USA 91711

## Abstract

NADH-dependent persulfide reductase (Npsr) has been proposed to facilitate dissimilatory sulfur respiration by reducing persulfide or sulfane sulfur-containing substrates to H_2_S. The presence of this gene in the sulfate and thiosulfate-reducing *Archaeoglobus fulgidus* DSM 4304 and other hyperthermophilic *Archaeoglobales* appears anomalous, as *A. fulgidus* is unable to respire S^0^ and grow in the presence of elemental sulfur. To assess the role of Npsr in the sulfur metabolism of *A. fulgidus* DSM 4304, the Npsr from *A. fulgidus* was characterized. AfNpsr is specific for persulfide and polysulfide as substrates in the oxidative half-reaction, exhibiting *k*_cat_/*K*_m_ on the order of 10^4^ M^−1^ s^−1^, which is similar to the kinetic parameters observed for hyperthermophilic CoA persulfide reductases. In contrast to the bacterial Npsr, AfNpsr exhibits low disulfide reductase activity with DTNB; however, similar to the bacterial enzymes, it does not show detectable activity with CoA-disulfide, oxidized glutathione, or cystine. The 3.1 Å X-ray structure of AfNpsr reveals access to the tightly bound catalytic CoA, and the active site Cys 42 is restricted by a flexible loop (residues 60-66) that is not seen in the bacterial homologs from *Shewanella loihica PV-4* and *Bacillus anthracis*. Unlike the bacterial enzymes, AfNpsr exhibits NADH oxidase activity and also shows no detectable activity with NADPH. Models suggest steric and electrostatic repulsions of the NADPH 2′-phosphate account for the strong preference for NADH. The presence of Npsr in the nonsulfur-reducing *A. fulgidus* suggests that the enzyme may offer some protection against S^0^ or serve in another metabolic role that has yet to be identified.

## 1. Introduction

Pyridine nucleotide disulfide oxidoreductases (PNDORs) are a large class of homodimeric NADH- and FAD-dependent enzymes that generally function to reduce small molecule substrates at a catalytic cysteine positioned at the si face of the isoalloxazine ring. The substrate preference and reactivity vary greatly depending on surrounding catalytic residues and additional structural elements, but broadly speaking, each subunit contributes catalytic residues to the active site. Two closely related PNDORs, CoA-disulfide reductase (CoADR) and NAD(P)H-dependent persulfide reductase (Npsr), reduce S^0^ and facilitate dissimilatory sulfur respiration in prokaryotes and archaea by catalyzing the reduction of either elemental sulfur, persulfides, or polysulfide to hydrogen sulfide.

(1) $$\begin{array}{c} {\text{S}}^{0},{{\text{S}}_{\text{n}}}^{2-}\text{ or CoA}-{\text{S}}_{\text{n}}-\text{SH}+\text{NAD}\left(\text{P}\right)\text{H}+{\text{H}}^{+}\to {\text{H}}_{2}\text{S}+\text{NAD}{\left(\text{P}\right)}^{+}+\text{CoA}-\text{SH or }{{\text{S}}_{\text{n}-1}}^{2-} \end{array}$$

**Scheme 1:**
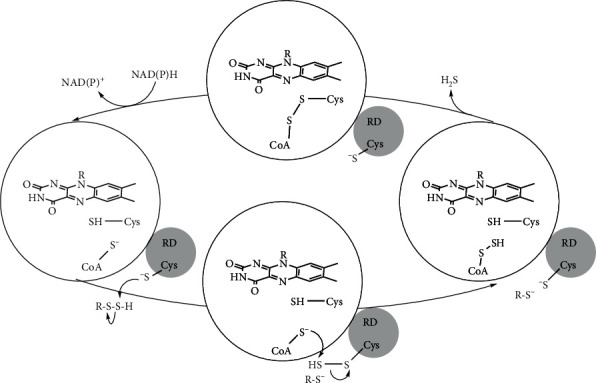
Reaction cycle.

Typically, there is a strong correlation between the presence of genes for these proteins and the ability of bacterial and archaeal species to respire by carrying out *in vivo* S^0^ reduction. Sulfur-reducing prokaryotes harboring these enzymes often participate in cycling sulfur and carbon in anoxic environments. The major structural difference between CoADR and Npsr is the addition of a ~100-amino acid rhodanese-like domain to the C-terminus of the Npsr [1, 2]. A surface-accessible cysteine in this domain serves as the initial sulfur-accepting site before a molecule of CoA bound to the FAD-containing domain chaperones the sulfur to the active site cysteine adjacent to FAD (Scheme 1). CoADR is widely distributed throughout thermophilic and hyperthermophilic archaea known to reduce and/or respire on S^0^. By contrast, Npsr is not found typically in archaea with the exception of Npsr homologs identified in the genomes of Archaeoglobales, which are dissimilatory sulfate-reducing (hyper) thermophiles [3]. More recently, microbial community analysis and sequencing have identified several new and uncharacterized archaeal species carrying genes for Npsr homologs. These include members of Crenarchaeota, Bathyarchaeota, Heimdallarchaeota, Haloarculaceae, Lokiarchaeota, Thermoplasmata, Methanomicrobiales, Verstraetearchaeota, and Methanomassiliicoccales.

Unlike the other CoADR and Npsr-containing organisms, Archaeoglobales cannot respire S^0^ and are actually inhibited by it [4]. Sequence homology to the bacterial Npsrs from *Shewanella oneidensis* PV4 and *Bacillus anthracis* suggests the homolog in Archaeoglobales could be a per/polysulfide reductase; however, the lack of a sulfur-reducing phenotype among the different species suggests the enzyme may have a new function, a different substrate preference, and/or altered catalytic properties [1, 5].

Given the sulfur intolerance of Archaeoglobales, the presence of Npsr in the genome is an oddity. To understand the function of this particular enzyme in the sulfur metabolism of *A. fulgidus* DSM 4303 and biogeochemical sulfur cycling, kinetic and structure data on the recombinant protein were gathered. Here, we show that the enzyme strongly prefers persulfide and polysulfide substrates over disulfides and has an unusual NADH oxidase activity and unique structural characteristics, suggesting a new as of yet unidentified role for this enzyme in the metabolism of a dissimilatory sulfate-reducing Achaean.

## 2. Materials and Methods

### 2.1. Expression and Purification of *A. fulgidus* Npsr

A codon optimized version the *A. fulgidus* Npsr gene (WP_010877907.1) was synthesized by GenScript and subcloned into a pET21b+vector using NdeI and XhoI restriction sites. The final construct introduced a C-terminal His_6_-tag. The optimized gene sequence can be found in the supporting information (Figure S1).

A starter culture of transformed BL21 (DE3) *E. coli* cells was grown overnight in 100 mL of terrific broth (TB) medium (12 g/L tryptone, 24 g/L yeast extract, 4 mL/L glycerol, 9.4 g/L KH_2_PO_4_, and 2.2 g/L K_2_HPO_4_) and 100 *μ*g/mL ampicillin at 37°C with 200 rpm shaking. The following day, 1 L of TB medium containing 100 *μ*g/mL ampicillin was inoculated and incubated as before. At an OD_600_ of 0.6-0.8, expression was induced by addition of 200 *μ*M IPTG. After 3-4 additional hours of shaking at 37°C, the cells were pelleted by centrifugation at 5000 × g and stored at -80°C until further use.

To purify the protein, the cells were thawed and resuspended in wash buffer (50 mM N_2_HPO_4_, pH 7.4, 200 mM NaCl, and 20 mM imidazole) supplemented with 1 mM MgCl_2_, DNase, and PMSF. The cells were lysed on ice by sonication at 400 W using 20 s pulses followed by 10 s rest over 8-10 min. The lysed cells were centrifuged at 10,000 × g for 10 min, after which 50 mg of FAD was added to the supernatant and heated in a 70°C water bath for 10 min to reconstitute the protein. The sample was centrifuged again at 40,000g for 45 min. The supernatant was removed, passed through a 0.22 *μ*m filter, and loaded onto a Ni^2+^-affinity column preconditioned in wash buffer. The column was washed with 10 column volumes of wash buffer followed by 4 column volumes of an elution buffer containing 50 mM N_2_HPO_4_, pH 7.5, 200 mM NaCl, and 300 mM imidazole, pH 7.4. The protein was concentrated to ~2 mL using a spin filter with a 100 kDa MW cutoff and purified further with Superdex 200 size exclusion in 25 mM Tris, pH 7.5, 100 mM NaCl, 1 mM *β*-mercaptoethanol, and 5% glycerol. SDS-PAGE was used to assess purity. The protein was concentrated to 10-30 mg/mL using a Millipore spin concentrator, flash frozen in liquid nitrogen, and stored at −80°C until further use. The extinction coefficient of *ε*_450_ = 11,300 M^−1^ cm^−1^ was used to estimate the protein concentration by UV-vis spectroscopy.

### 2.2. Crystallization, Data Collection, and Structure Determination

*Af*Npsr was thawed and exchanged into 25 mM Tris, pH 7.5, 25 mM NaCl using a centrifugal concentrator. The protein was crystallized by using the hanging and sitting drop vapor diffusion methods at room temperature by combining 2 *μ*L of protein at 7 mg/mL with 2 *μ*L of a precipitant solution comprising 100-200 mM calcium acetate, 15-20% PEG 3350 (*w*/*w*), and 1 mM DTT. Rectangular plates appeared within 2-5 days. Prior to data collection, the crystals were transferred to a cryosolution containing 200 mM calcium acetate, 20% PEG 3350 (*w*/*w*), and 20% glycerol and flash frozen in liquid nitrogen. Data were collected at 100 K at the Advanced Photon Source (APS) on NE-CAT beamline 24-ID-E.

The crystals belonged to the space group P12_1_1 with unit cell dimensions *a* = 84.98 Å, *b* = 100.36 Å, *c* = 136.20 Å and *α* = 90.0°, *β* = 91.9°, *γ* = 90.0°. The data were integrated by using XDS then merged and scaled using AIMLESS from the CCP4 suite of programs (Table 1) [6, 7]. The initial phases were determined by molecular replacement using BALBES [8]. The best solution was obtained using 3NT6 as the starting model. The asymmetric unit (ASU) contained two AfNpsr dimers or four total subunits. The model of *Af*Npsr was built with Coot, and refinements were carried out with Phenix using NCS constraints and TLS parameters (Table 1) [9, 10]. A Ramachandran plot calculation indicated that 94.2% and 5.5% of the residues occupy the most favored and additionally allowed regions, respectively.

### 2.3. Synthesis of Persulfide Substrates

Oxidized glutathione, cystine, CoA-disulfide, and sodium sulfide were purchased from Sigma-Aldrich Corp. (St. Louis, MO), solubilized in 100 mM sodium phosphate buffer, pH 8, and made anaerobic by sparging a stoppered vial with N_2_ on a Schlenk line. Using a syringe, 2-fold excess sodium sulfide was added to each oxidized disulfide compound, creating final stock solutions of 5 mM cysteine persulfide, 10 mM glutathione persulfide, and 2 mM CoA-persulfide. Stocks of the persulfide substrates were made fresh daily.

### 2.4. Synthesis of Polysulfide

Equimolar concentrations of sulfur (56.4 g) and sodium sulfide (54.1 g) (Sigma) were added to 100 mL of boiling H_2_O and allowed to react for ~15 min with stirring to create a 2.25 M polysulfide stock solution. When the reaction turned dark orange, the solution was cooled and transferred to a sterile bottle with minimal headspace.

### 2.5. Steady-State Kinetic Assays

Assays were conducted aerobically on an Agilent UV-vis spectrophotometer fitted with Peltier temperature control and conducted in quartz cuvettes in a total volume of 1 mL. Each assay was performed at 70°C and contained 100-200 nM of enzyme in 50 mM sodium phosphate buffer, pH 7.5, and 100 *μ*M NAD(P)H unless otherwise stated. Assays with polysulfide were performed in 1 M Tris at pH 8.7 to stabilize the substrate and prevent changes in pH due to the addition of the basic polysulfide solution [1, 11]. All buffers were prepared at room temperature. The activity for substrates other than DTNB was monitored by observing the oxidation of NAD(P)H at 340 nm (*ε* = 6220 M^−1^ cm^−1^). DTNB (5,5′dithiobis-2-nitrobenzoic acid) (Sigma) reductase activity was monitored at 412 nm (TNB (5-thio-2-nitrobenzoic acid) *ε* = 14,150 M^−1^ cm^−1^).

Before the start of each assay, thiol substrates and enzyme were preincubated at 70°C in the cuvette. Each assay was initiated by the addition of NAD(P)H. The rate of background NAD(P)H oxidation was recorded under each condition and subtracted when appropriate. For reactions monitoring the reduction of DTNB, the background rate of TNB formation in buffer was subtracted from the observed rate. The kinetic constants were determined by performing a nonlinear fit to the Michaelis-Menten equation.

### 2.6. Anaerobic Assays for Specific Activity

Assays for specific activity were performed in a Coy anaerobic chamber. All stock solutions were made anaerobic by sparging with N_2_ on a Schlenk line. Each 1 mL reaction comprising 0.1 *μ*M enzyme, 160 *μ*M NADH, and 200 *μ*M persulfide or polysulfide substrate in 1 M Tris, pH 8.7, was performed in a 70°C heat block. The reaction was initiated by the addition of NADH and stopped after 1 min by placing the reaction in a chiller block. The activity was assessed by comparing the absorbance at 340 nm of samples with and without the enzyme present. Each reaction was performed in triplicate.

## 3. Results

### 3.1. Steady-State Kinetics

Under steady-state conditions, AfNpsr showed a preference for persulfide and polysulfide substrates, exhibiting *k*_cat_s between 10 and 230 s^−1^ and specificity constants on the order of 1 × 10^4^ M^−1^ s^−1^ (Table 2). Of the sulfur substrates tested, cysteine persulfide and polysulfide were preferred over glutathione and CoA persulfides, as evidenced by the higher turnover numbers and ~3-fold higher *k*_cat_/*K*_m_. AfNpsr demonstrated the least activity toward DTNB and had no detectable reaction with the disulfide substrates cystine, oxidized glutathione, and CoA-disulfide. These observations suggest disulfides have restricted access to the AfNpsr active site. CoADR from *Pyrococcus horikoshii*, which has 40% sequence identity and 56% similarity to AfNpsr, has similar substrate preference toward per- and polysulfides over disulfides [12].

**Figure 1:**
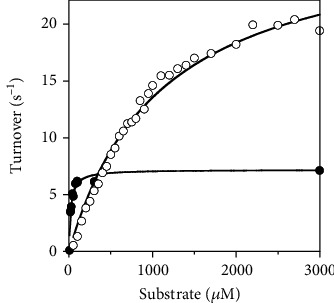


Assays assessing the NADH oxidase chemistry of the enzyme revealed AfNpsr is specific for NADH and shows no detectable activity with NADPH, suggesting AfNpsr restricts NADPH binding by selecting against the 2′ phosphate (vide infra). Under aerobic conditions, AfNpsr demonstrated reasonable NADH oxidase activity, with a *k*_cat_ that is a little more than half that of CoA persulfide, the substrate with the lowest turnover number (Figure 1 and Table 2). Typically, when assessing the activity of PNDOR enzymes under aerobic conditions, the rate for the background NADH oxidase activity is subtracted from the rate determined for the reduction of substrates. For AfNpsr, however, the rate of di, per, and polysulfide reduction at low concentrations of substrate (as measured by monitoring NADH consumption) was less than the rate of the NADH oxidase reaction in the absence of substrates (Figure 1). For example, at 160 *μ*M NADH, the turnover number for the NADH oxidase reaction was ~6 s^−1^. Upon adding 50-100 *μ*M GSH persulfide to assays containing 160 *μ*M NADH, the turnover number drops to ~1 s^−1^. Assuming 100% air saturation at 70°C, the estimated concentration of dissolved O_2_ in each assay is 120 *μ*M [13]. Under assay conditions in which the O_2_ concentration was higher than the concentration of per/polysulfide substrates, the rate of NADH consumption was lower in comparison to the rate observed in the absence of substrates (i.e., the NADH oxidase reaction). Because the NADH consumption rate *decreases* in the presence of per/polysulfide substrates, we infer that these substrates are effectively outcompeting O_2_ for access to the active site. As such, for these assays, the background oxidase rates were not subtracted, as the resulting rates would be negative. It cannot be ruled out, however, that the observed rates with substrate include some amount of NADH oxidase activity.

### 3.2. Anaerobic Assay of Specific Activity

As a control to assess substrate turnover in the absence of O_2_ and to confirm that the experiments conducted under aerobic conditions are a reasonable representation of the reaction of the enzyme with the substrates, the specific activity of the enzyme with the different per- and polysulfide substrates was measured under anaerobic conditions at 70°C (Table 3). Under these conditions, no NADH oxidase activity was observed, and all 4 persulfide/polysulfide substrates showed significant activity. While we have reported the values obtained from these assays, due to the nature of the assay (especially the less than optimal but technically necessary method of stopping the reaction on a chiller block), these rates are sufficiently inaccurate that direct comparison of them to the standard assay would likely result in overinterpretation of the results—they are meant more to serve as qualitative assays confirming the lack of NADH oxidase activity and presence of per/polysulfide reductase activity under anaerobic conditions.

### 3.3. Global Structure of AfNpsr

**Figure 2:**
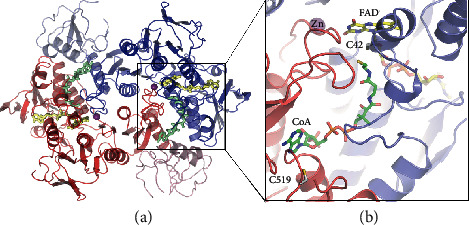


The final model of AfCoADR was refined to 3.1 Å and had *R* and *R*_free_ of 19.0% and 25.8%, respectively (Table 1). As expected, the enzyme is a dimer, with the N-terminal FAD-binding domain (residues 1-448) having nearly identical topology to related Npsr and CoADR proteins (Figure 2). The r.m.s.d. between *A. fulgidus* and *Shewanella loihica* NPSR, which are 35% identical and 54% similar, is 1.66 Å^2^. By comparison, the r.m.s.d. between AfNpsr and *Pyrococcus horikoshii* CoADR, which are 40% identical and 55% similar, is 1.81 Å^2^. Residues 449 through 550 make up the C-terminal rhodanese domain, which is linked to the FAD domain by a single amino acid chain. On all four AfNpsr subunits found in the asymmetric unit, two loops on the rhodanese domain comprising residues 465-472 and 483-488 were missing due to disorder.

### 3.4. Substrate Access to the Active Site

**Figure 3:**
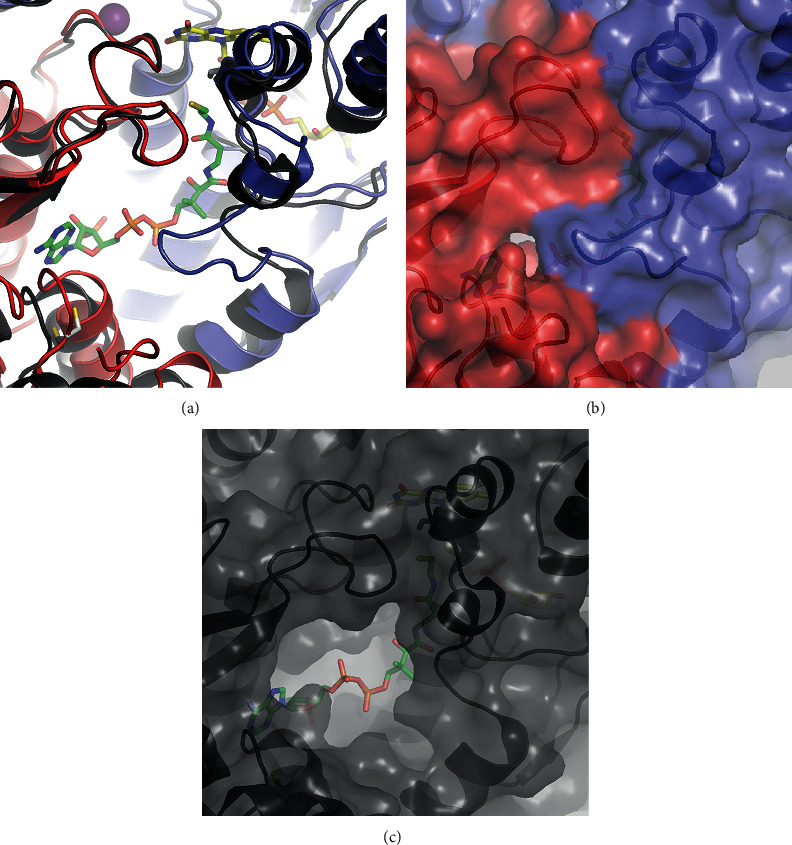


Each AfNpsr subunit and part of a rhodanese domain contributes to the formation of the CoA binding pocket (Figures 2 and 3). In AfNpsr, CoA is deeply buried, and in this configuration of the protein, CoA has little access to the solvent except for a small opening above the pantothenic acid arm (Figures 3(a) and 3(b)). Located near this opening is Cys 519 of the rhodanese domain, which has been proposed to help shuttle sulfide substrates to CoA. By contrast, the CoA binding pocket in the *Shewanella loihica* and *Bacillus anthracis* Npsr structures is more open and perhaps more readily allows reduced CoA to swing out and grab potential substrates bound to the rhodanese domain cysteine (Figure 3(c) and Scheme 1) [1, 5].

In AfNpsr, the seemingly low accessibility of CoA from the surface of the protein results from a larger loop consisting of residues 60-66 (TTYGAVR) that closes over the CoA binding pocket (Figure 3(a)). On one of the dimers in the asymmetric unit, this loop is ordered in both subunits (Supplemental Figure 3A). On the second dimer, residues 62-65 in both subunits are disordered and cannot be modeled (Supplemental Figure 3B). It should be noted that for the dimer with the ordered loop, the average B-factor for the loop is 67 Å^2^ compared to 47 Å^2^ for the rest of the FAD-binding domain, suggesting there is some flexibility for the opening and closing of the loop over the enzyme-bound CoA. In structures of CoADRs and other PNDOR enzymes like NADH oxidase (Nox), peroxidase (Npx), and glutathione reductase (GR), this loop does not exist [14–17].

### 3.5. Structural Basis for Preference of NADH over NADPH

**Figure 4:**
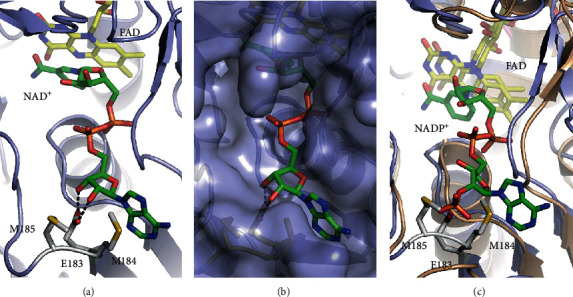


The steady-state kinetics indicated AfNpsr is only active when NADH is the cosubstrate. To explain the strong preference for NAD^+^ over NADP^+^, both cofactors were manually docked onto the enzyme with the aid of existing crystal structures of PNDOR proteins in which these compounds are bound. The model with bound NAD^+^ shows the cofactor fitting snugly into the NADH-binding pocket and suggests the ribose 2′ and 3′-hydroxyl moieties hydrogen bond to E183 (Figure 4). For NADP^+^, the phosphorylated 2′ hydroxyl group of ribose introduces steric clashes with E183, M184, and M185 as well as strong electrostatic repulsions with E183. The models suggest steric and electrostatic repulsions likely disfavor NADPH binding and its use as a cofactor by AfNpsr.

### 3.6. Metal Binding Site near FAD

A metal ion binding site is located 5.2 Å from FAD (Figure 2, Supplemental Figure 2). This site is removed from the active site and appears to be unique to AfNpsr, as it has never been observed in any PNDOR family member. Presently, the identity of the bound metal is not clear, but several lines of evidence suggest Zn^2+^ is an appropriate tentative assignment. First, UV-vis spectroscopy does not show any additional absorption bands beyond those belonging to FAD, ruling out paramagnetic metal ions. Second, fluorescence scans of the K-edge for Fe, Ni, Co, Cu, and Zn carried out on AfNpsr crystals using X-ray diffraction beamline 12-2 at SSRL revealed a small fluorescence peak for Zn. Despite several attempts, anomalous diffraction data at the zinc K-edge were not taken because the crystal quality was poor. Modeling Zn^2+^ into the metal site accounts for most of the difference density as opposed to fitting with lighter atoms like Ca^2+^ and Mg^2+^. An assignment of Zn^2+^ is also consistent with an oxygen-rich coordination sphere and the pseudooctahedral geometry. Although the coordination number appears low (4), there may be additional solvent-derived ligands coordinating to the metal that cannot be observed because of the low resolution of the structure. In light of finding this metal binding site, activity assays were conducted with small concentrations (0.5-5 mM) of different metal ions. In all cases, no observable increase in activity was detected (data not shown). Given that AfNpsr has all of the same catalytic components as the other Npsr and CoADR homologs and that none of these have any demonstrated metal dependence, it is likely that the bound metal ion in AfNpsr plays a structural role.

## 4. Discussion

*Archaeoglobus fulgidus* is a hyperthermophilic archaeon isolated from hydrothermal vents and oil fields that can survive in anaerobic environments approaching temperatures of 95°C. Although the genus is most closely related to methanogens and carries most of the enzymes that allow for methanogenesis, it lacks the terminal step and necessary cofactors for methane biosynthesis. Instead, Archaeoglobales use sulfate and sulfite as terminal electron acceptors [4]. Although cultivation studies indicate that *A. fulgidus* is intolerant of elemental sulfur, the kinetic data presented above indicate that *A. fulgidus* carries a gene for a functional Npsr that can facilitate dissimilatory S^0^ reduction by reducing per- and polysulfides to H_2_S. Moreover, genes for two putative CoADRs are encoded in the *A. fulgidus* genome, and preliminary work in our laboratory has confirmed that at least one of these has disulfide reductase activity (unpublished result). Thus, the presence of S^0^-reducing proteins in Archaeoglobales provides a puzzle as to their biological function in these archaea.

A transcriptome analysis of *A. fulgidus* comparing heterotrophic growth on lactate to lithoautotrophic growth with H_2_/CO using thiosulfate and sulfate as the terminal electron acceptors sheds some light onto the general metabolic role of Npsr [18]. Under all growth conditions, including samples taken in both the log and late log phases of growth, the average transcriptional abundance of AfNpsr was 2.2- to 3.4-fold greater than the mean of all transcripts. Transcripts of AfNpsr were almost 2-fold higher under lithoautotrophic growth conditions than heterotrophic conditions. These findings indicate the protein is translated and regularly contributes in some way to sustaining *A. fulgidus* regardless of the growth phase. One possible role for the AfNpsr may be in the reduction of S^0^ that can form during metabolism, as production of S^0^ globules during the reduction of thiosulfate by anaerobic hyperthermophiles has been previously observed [19, 20]. Because *A. fulgidus* is not able to grow in the presence of high concentrations of S^0^, this enzyme may play a role in preventing the formation of sulfur globules in or near the cells.

*A. fulgidus* Npsr displayed significant glutathione persulfide, cysteine persulfide, CoA persulfide, and polysulfide reductase activities, with *k*_cat_/*K*_m_ values on the order of 10^4^ M^−1^ s^−1^, which is high enough to suggest the physiological substrate for AfNpsr may be a persulfide or polysulfide compound. While the enzyme was able to reduce DTNB, it showed no catalytic activity toward the CoA and glutathione disulfides or cystine, indicating these substrates are simply too large for access to the either the rhodanese cysteine located near the surface (C519) or the enzyme-bound CoA. The substrate preference of AfNpsr for persulfides and polysulfides over disulfides is consistent with those of the characterized Npsr homolog from *S. loihica* [1]. The restricted access to the active site-bound CoA, as compared to SlNpsr and BaNpsr, suggests perhaps that AfNpsr is more selective for smaller metabolites [1, 5]. Although it is unclear what the physiological substrate of AfNpsr may be, *A. fulgidus* is unlikely to contain glutathione, as its genome does not contain either of the glutathione synthetic enzymes (glutamine-cystine ligase or glutathione synthetase) or glutathione reductase (as determined by BLAST). Moreover, cysteine levels are expected to be kept at low levels due to its cytotoxic nature [21]. Their use by the enzyme suggests that CoA persulfide and polysulfides may be a more viable substrate candidates.

Because *A fulgidus* Npsr exists within a family of proteins with a diverse range of functions and substrates, its own role therefore could vary beyond the simple reduction of per/polysulfides or disulfides. The per/polysulfide reductase activity of AfNpsr may suggest the enzyme has a protective role, acting in either an antioxidant or detoxification capacity. AfNpsr transcripts being present at higher levels than most other transcripts in both the log and late log phases of growth is consent with a housekeeping function [18]. Likewise, the NADH oxidase reaction presents the possibility that Npsr could help the organism mitigate O_2_ stress; however, it is unclear whether the NADH oxidase activity is a true moonlighting function of the enzyme. Such a function is not without precedent, as a deletion of the gene encoding CoADR in *Thermococcus kodakarensis* demonstrated increased sensitivity to oxygen in the presence of sulfur, while its sensitivity to oxygen in the absence of sulfur remained the same [22]. Although AfNpsr has *in vitro* polysulfide reductase activity, the poor growth on S^0^ clearly indicates the *in vivo* activity is not sufficient to confer Archaeoglobales resistance to the toxic effects of large amounts of S^0^ or an ability to respire it.

## 5. Conclusions

*A. fulgidus* Npsr displays structural similarity to other CoADR/NPSR proteins, with a conserved active site and cofactors. Its rhodanese tail resembles the domain found in *S. loihica* Npsr but is absent from CoADRs. This and differences in the loop closing over the active site CoA potentially account for the differences in substrate specificity. *A. fulgidus* Npsr displays affinity for reduction of persulfide and polysulfide substrates and an exclusive preference for NADH over NADPH as the reductant, possibly providing it a biological role as a producer of NAD^+^ to maintain a pool of electron acceptors for metabolism. Further work to examine the functions of the related AfCoADR proteins and the response of Archaeoglobales to S^0^ is required to clarify how Archaeoglobales manage this type of metabolic stress or repurpose these enzymes for an as yet unidentified function.

## Figures and Tables

**Table 1:** X-ray data collection and refinement statistics for *Af*Npsr.

*Data collection*	
Beamline	NE-CAT 24-ID-E
Wavelength (Å)	0.98
Space group	*P12_1_1*
Unit cell dimensions (Å)	*a* = 84.98, *b* = 100.36 Å, *c* = 136.20 Å *α* = 90.0°, *β* = 91.9°, *γ* = 90.0°
Resolution range (Å)	40-3.1 (3.27-3.10)
Total reflections	153,972 (22,435)
Unique reflections	41,638 (6083)
Completeness (%)^a^	99.7 (99.9)
CC 1/2	99.2 (76.1)
Multiplicity	5.2 (4.8)
*I*/*σ* (*I*)^a^	10.3 (2.6)
Rsym_sym_ (%)^a,b^	11.9 (54.0)
*Refinement*	
*R*_cryst_ (%)^c^	19.0
*R*_free_ (%)^d^	25.8
r.m.s. deviation bond length (Å)	0.021
r.m.s. deviation bond angles (°)	1.998
Dimers per ASU	2
No. protein atoms	16,267
No. nonprotein atoms	422
Water molecules	14
PDB code	6PFZ

^a^Values in parentheses are for the highest resolution shell. ^b^*R*_sym_ = Σ_*i*_Σ_*hkl*_ | *I*_*i*_(*hkl*) − <*I*(*hkl*) > ∣/Σ_*hkl*_ < *I*(*hkl*)>, where *I*_*i*_(*hkl*) is the *i*th measured diffraction intensity and <*I*(*hkl*)> is the mean intensity for the Miller index (*hkl*). ^c^*R*_cryst_ = Σ_*hkl*_‖*F*_o_(*hkl*) | −∣*F*_c_(*hkl*)‖/Σ_*hkl*_ | *F*_o_(*hkl*)∣. ^d^*R*_free_ = *R*_cryst_ for a test set of reflections (5% in each case).

**Table 2:** Steady-state kinetics of AfNpsr under aerobic conditions at 70°C.

Substrate^a^	*k*_cat_ (s^−1^)	*K*_m_ (mM)	*k*_cat_/*K*_m_ (M^−1^ s^−1^)
NADH^b^	7.15 ± 0.51	0.0206 ± 0.0051	3.47 ± 0.75 × 10^5^
NADPH^b^	NR^c^	—	—
DTNB	2.44 ± 0.14	0.743 ± 0.196	3.28 ± 0.48 × 10^3^
Coenzyme A disulfide	NR	—	—
Glutathione disulfide	NR	—	—
Cystine	NR	—	—
Coenzyme A persulfide	13.2 ± 0.3	0.547 ± 0.041	2.41 ± 0.14 × 10^4^
Glutathione persulfide	29.8 ± 1.2	1.25 ± 0.23	2.44 ± 0.49 × 10^4^
Cysteine persulfide	230 ± 23	3.41 ± 0.63	6.76 ± 1.1 × 10^4^
Polysulfide^d^	42.0 ± 2.2	0.616 ± 0.058	6.82 ± 0.29 × 10^4^

^a^All reactions performed with di-, per-, and polysulfide substrates contained 100 *μ*M NADH. ^b^NAD(P)H oxidase activity. No di-, per-, or polysulfides were present. ^c^NR: no reaction. ^d^The reaction was performed in 1 M Tris, pH 8.7, as in (8).

**Table 3:** Specific activity of AfNpsr under anaerobic conditions at 70°C.

Substrate	Specific activity (min^−1^)
NADH	NR^a^
Cysteine persulfide	2.25 ± 0.68 × 10^2^
Glutathione persulfide	9.86 ± 0.65 × 10^2^
CoA persulfide	1.45 ± 0.13 × 10^3^
Polysulfide^b^	1.32 ± 0.04 × 10^3^

^a^NR: no reaction. ^b^The reaction was performed in 1 M Tris, as in (8).

## Data Availability

The coordinates and structure factors for *Archaeoglobus fulgidus* Npsr have been deposited in the Protein Data Bank (http://www.rcsb.org) as entry 6PFZ (https://www.rcsb.org/structure/6PFZ).

## Abbreviations

AfNpsr: *Archaeoglobus fulgidus* NAD(P)H-dependent persulfide reductase
CoADR: Coenzyme A-disulfide reductase
phCoADR: *Pyrococcus horikoshii* coenzyme A-disulfide reductase
DTNB: 5,5′-Dithiobis-2-nitrobenzoic acid
PNDOR: Pyridine nucleotide disulfide oxidoreductase
Npsr: NAD(P)H-dependent persulfide reductase.

## Supplementary Materials

Supplementary Materials Figure S1: codon optimized *A. fulgidus* Npsr gene WP_010877907.1. Figure S2: metal binding site adjacent to FAD. Figure S3: composite omit electron density map of the ordered and disordered active site surface loop above coenzyme A.
