# Supplementary material for: Structural and Kinetic Characterization of Hyperthermophilic NADH-Dependent Persulfide Reductase from Archaeoglobus fulgidus
Source: Archaea. 2021 Mar 9;2021:8817136. doi: 10.1155/2021/8817136 (PMC7969121; doi:10.1155/2021/8817136)
Supplement: Supplementary Materials — Figure S1: codon optimized A. fulgidus Npsr gene WP_010877907.1. Figure S2: metal binding site adjacent to FAD. Figure S3: composite omit electron density map of the ordered and disordered active site surface loop above coenzyme A. [file 8817136.f1.zip › 8817136.f2.docx]

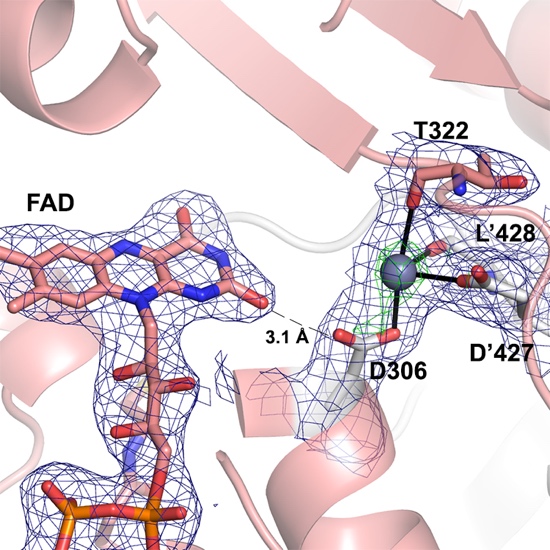


**Figure S2.** Metal binding site adjacent to FAD. 2F_o_-F_c_ electron density maps are contoured to 1σ (blue) and 5σ (green).
