# Supplementary material for: Structural and Kinetic Characterization of Hyperthermophilic NADH-Dependent Persulfide Reductase from Archaeoglobus fulgidus
Source: Archaea. 2021 Mar 9;2021:8817136. doi: 10.1155/2021/8817136 (PMC7969121; doi:10.1155/2021/8817136)
Supplement: Supplementary Materials — Figure S1: codon optimized A. fulgidus Npsr gene WP_010877907.1. Figure S2: metal binding site adjacent to FAD. Figure S3: composite omit electron density map of the ordered and disordered active site surface loop above coenzyme A. [file 8817136.f1.zip › 8817136.f3.docx]

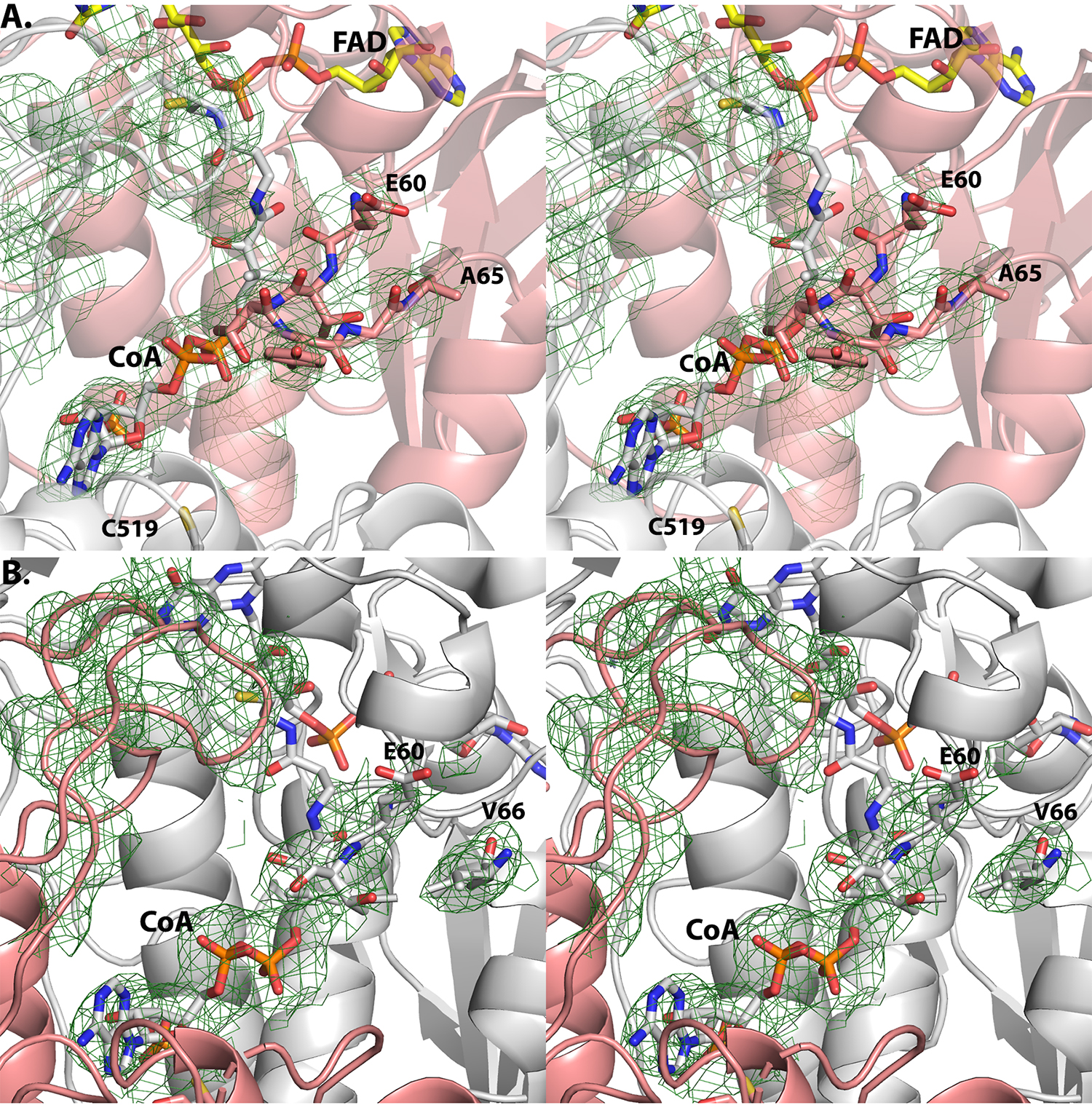


**Figure S3.** 2Fo-Fc composite omit electron density map contoured to 1σ of the (A) ordered and (B) disordered active site surface loop (residues 60-65) above coenzyme A in stereo.
